# Supplementary material for: Noninvasive Subcellular Imaging Using Atomic Force Acoustic Microscopy (AFAM)
Source: Cells. 2019 Apr 5;8(4):314. doi: 10.3390/cells8040314 (PMC6523517; doi:10.3390/cells8040314)
Supplement: Supplementary file 1 [file cells-08-00314-s001.pdf]

cells

MDPI

Supporting Information

Noninvasive subcellular imaging using scanning probe acoustic microscopy

Xiaoqing Li[1], Wenjie Deng[1], Ang Lu[2], Li Su[2], Jing Wang[1,*] and Mingyue Ding[1,*]

[1] Depatrment of Biomedical Engineering, College of Life Science and Technology, Huazhong University of Science and Technology, Wuhan, 430074, China; d201677426@hust.edu.cn (X. L.), 782370615@qq.com (W. D.)

[2] Depatrment of Biophysics, College of Life Science and Technology, Huazhong University of Science and Technology, Wuhan, 430074, China; 348411351@qq.com (A. L.), lisu@hust.edu.cn (L. S.)

* Correspondence:wang.jing@hust.edu.cn (J. W.); myding@hust.edu.cn (M. D.)

Supporting results: Figure S1-S3

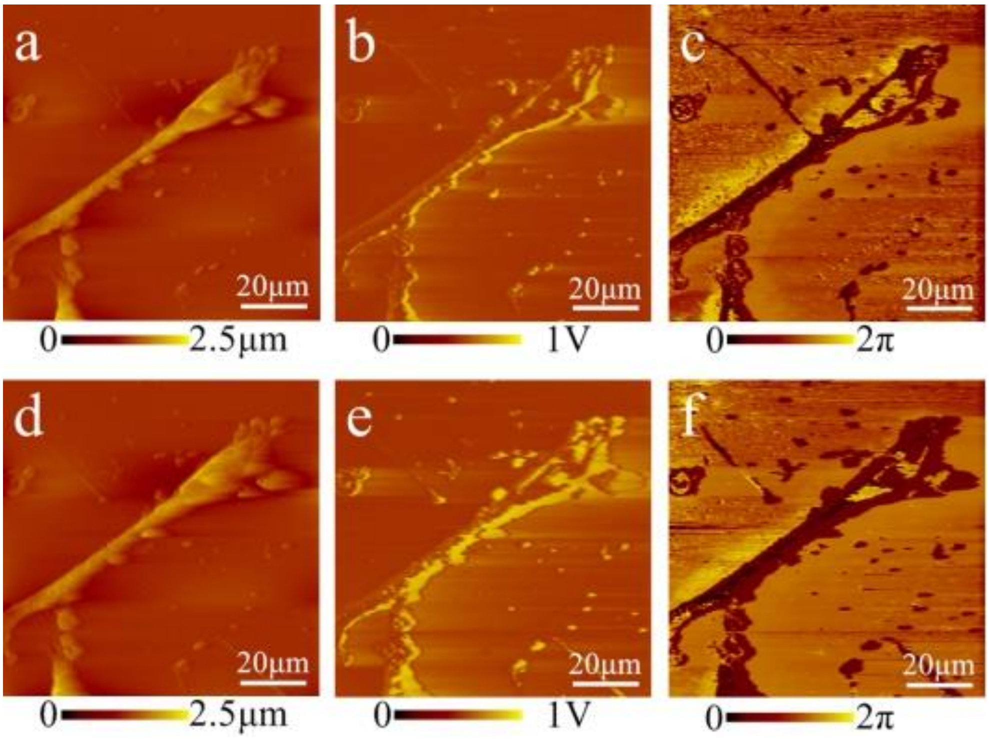

Figure S1. Optimization of the scanning speed for SPAM images of MDA-MB-231 cells. (a-c) The morphological image, the ultrasonic amplitude image and the phase image of a MDA-MB-231 cell obtained at a scanning speed of 0.5 Hz. (d-f) The morphology, the ultrasonic amplitude image and the phase image obtained at a scanning speed of 2 Hz yielded rough edges and an unclear surface structure.

cells

MDPI

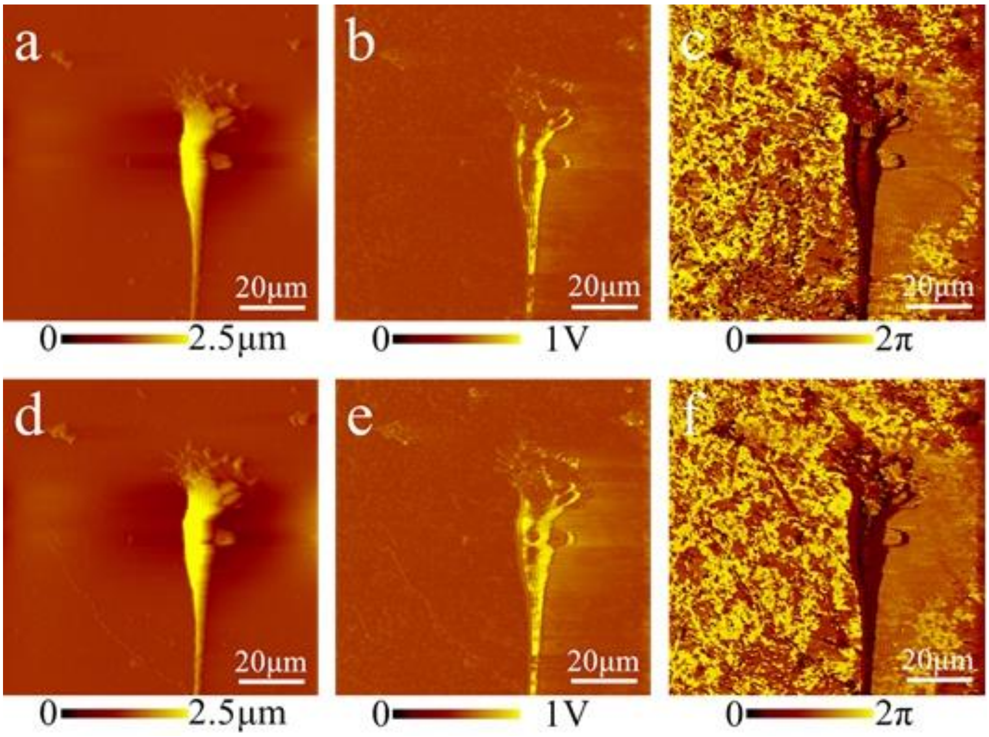

Figure S2. Optimization of feedback configurations for SPAM images of MDA-MB-231 cells. (a-c) The morphological image, the amplitude image and the phase image of an MDA-MB-231 cell obtained with an integral gain of 200 times, a proportional gain of 300 times and a reference point of 0.14 V. (d-f) The morphological image, amplitude image and phase image obtained at an integral gain of 300 times, a proportional gain of 200 times and a reference point value of 0.18 V showed rough edges with more artifacts. The parameter optimization of the feedback configurations improved the quality of images, with smoother edges and fewer artifacts.

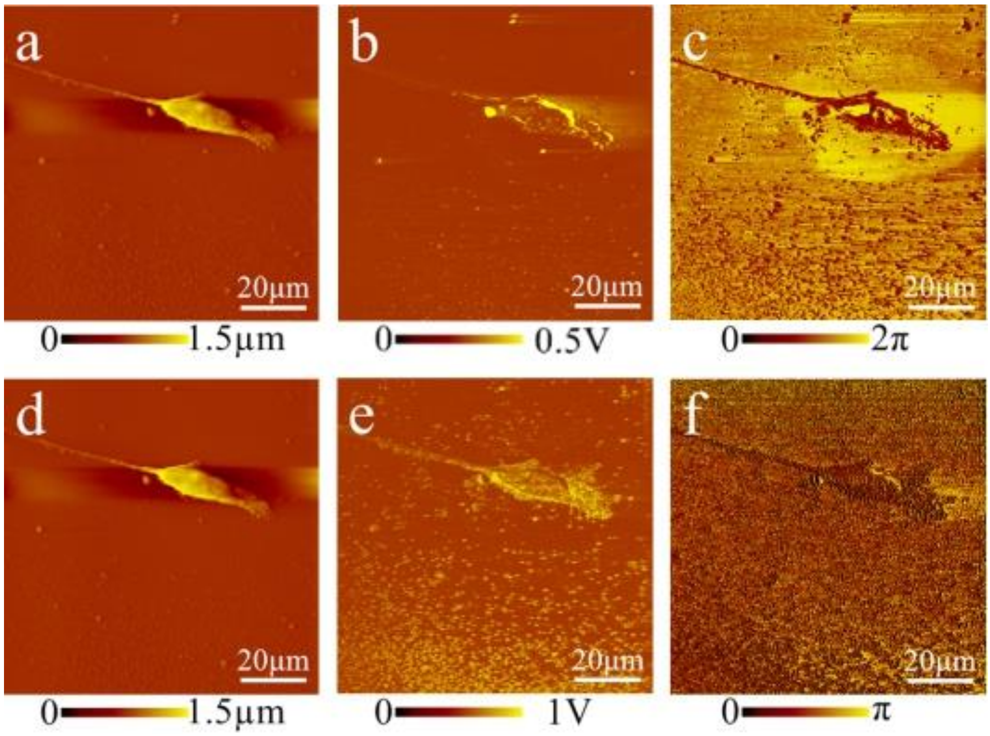

Figure S3. Optimization of acoustic frequency for SPAM images of MDA-MB-231 cells. (a-c) The morphological image, the amplitude image and the phase image of an MDA-MB-231 cell obtained at an acoustic frequency of 30.5 kHz. (d-f) The morphological image, the ultrasonic amplitude image and the phase image obtained at an acoustic frequency of 1.5 MHz had more artifacts and noise, but the morphological image did not substantially change.
